# Aberrant insulin signaling in Alzheimer's disease: current knowledge

**DOI:** 10.3389/fnins.2015.00204

**Published:** 2015-06-16

**Authors:** Gaurav Bedse, Fabio Di Domenico, Gaetano Serviddio, Tommaso Cassano

**Affiliations:** ^1^Department of Physiology and Pharmacology “V. Erspamer,” Sapienza University of RomeRome, Italy; ^2^Department of Biochemical Sciences, Sapienza University of RomeRome, Italy; ^3^Department of Medical and Surgical Sciences, University of FoggiaFoggia, Italy; ^4^Department of Clinical and Experimental Medicine, University of FoggiaFoggia, Italy

**Keywords:** insulin signaling, insulin-like growth factor, Alzheimer's disease, beta amyloid, phosphorylated tau

## Abstract

Alzheimer's disease (AD) is the most common form of dementia affecting elderly people. AD is a multifaceted pathology characterized by accumulation of extracellular neuritic plaques, intracellular neurofibrillary tangles (NFTs) and neuronal loss mainly in the cortex and hippocampus. AD etiology appears to be linked to a multitude of mechanisms that have not been yet completely elucidated. For long time, it was considered that insulin signaling has only peripheral actions but now it is widely accepted that insulin has neuromodulatory actions in the brain. Insulin signaling is involved in numerous brain functions including cognition and memory that are impaired in AD. Recent studies suggest that AD may be linked to brain insulin resistance and patients with diabetes have an increased risk of developing AD compared to healthy individuals. Indeed insulin resistance, increased inflammation and impaired metabolism are key pathological features of both AD and diabetes. However, the precise mechanisms involved in the development of AD in patients with diabetes are not yet fully understood. In this review we will discuss the role played by aberrant brain insulin signaling in AD. In detail, we will focus on the role of insulin signaling in the deposition of neuritic plaques and intracellular NFTs. Considering that insulin mitigates beta-amyloid deposition and phosphorylation of tau, pharmacological strategies restoring brain insulin signaling, such as intranasal delivery of insulin, could have significant therapeutic potential in AD treatment.

## Introduction

Alzheimer's disease (AD) is the most common cause of dementia. About 35.6 million people worldwide are now suffering from AD, and disease prevalence is expected to affect 115 million by 2050 (Wortmann, 2012). AD is a progressive, degenerative, and irreversible neurological disorder that causes deterioration of memory, judgment, and reasoning in the elderly (Querfurth and Laferla, 2010). Patients suffering from AD exhibit cognitive impairment, memory loss, and behavioral changes (Querfurth and Laferla, 2010). The neurodegeneration in AD is characterized by neuronal loss and synaptic injury (Dekosky and Scheff, 1990). The main pathological hallmarks of AD are extracellular insoluble beta amyloid (Aβ) plaques (Selkoe, 1989) and intracellular neurofibrillary tangles (NFTs). AD may be classified in two types based on genetic endowment. The first type is inherited via an autosomal dominant pattern, i.e., familial AD, and the second type is sporadic AD. Familial AD displays early disease onset, whereas sporadic AD cases mostly develop the disorder at an older age (Cruts and Van Broeckhoven, 1998). Although AD was discovered a century ago, the etiology of sporadic AD is not well understood. Over decades, it was hypothesized that neurodegeneration in AD is mainly caused by Aβ accumulation, phosphorylated tau aggregation, and/or neuroinflammation. However, recent human and preclinical studies have provided convincing evidence that AD is a degenerative metabolic disease, which is mediated by impairments in brain insulin responsiveness, glucose utilization, and energy metabolism leading to increased oxidative stress, inflammation, and worsening of insulin resistance (Hoyer, 2002, 2004; Schubert et al., 2004; Rivera et al., 2005; Steen et al., 2005; Watson and Craft, 2006; Craft, 2007; Neumann et al., 2008; Krikorian et al., 2010; Luchsinger, 2010; Baker et al., 2011; Talbot et al., 2012; Butterfield et al., 2014a,b; de La Monte, 2014).

The accumulating evidence that reduced glucose utilization and deficient energy metabolism occur early in the course of disease, suggests a role for impaired insulin signaling in the pathogenesis of neurodegenerative diseases. In brain, the insulin/insulin-like growth factor (IGF) signaling is important for neuronal growth, synaptic maintenance and neuroprotection (Stockhorst et al., 2004; Van Dam and Aleman, 2004). It is now proposed that impairments in brain insulin/IGF signaling is associated with increased accumulation of Aβ, phosphorylated tau, reactive oxygen/nitrogen species, pro-inflammatory and pro-apoptosis molecules (de La Monte et al., 2009; de La Monte, 2012a,b, 2014). Both restoration of insulin responsiveness and use of insulin therapy can lead to improved cognitive performance. This review focuses on the recent progress in our understanding of the neuronal insulin signaling in the pathogenesis and progression of AD. The review will also discuss therapeutic augmentation of brain insulin signaling by intranasal insulin delivery as a promising treatment for AD.

## Insulin signaling in the brain

The role of insulin in the brain has been little studied compared with its role in peripheral tissue. However, there is evidence that insulin has important functions in the brain including metabolic, neurotrophic, neuromodulatory and neuroendocrine actions. Recent studies have demonstrated that insulin and insulin receptor (IR) are ubiquitously expressed in the brain as in peripheral tissues (Schulingkamp et al., 2000; Van Der Heide et al., 2006). Brain insulin levels can reach 10- to 100- fold greater than in plasma, especially in hippocampus, hypothalamus, cortex, olfactory bulb, substantia nigra and pituitary (Frolich et al., 1998; Van Der Heide et al., 2006). IRs are abundantly expressed in neurons and less abundantly in glia (Frolich et al., 1998). Insulin is a hormone that has wide range of functions. Insulin is important for cell growth and survival. In the brain, insulin, IGFs and their receptors regulate dendritic sprouting, neuronal stem cell activation, cell growth, repair, synaptic maintenance and neuroprotection (Craft and Watson, 2004; Hoyer, 2004; Stockhorst et al., 2004; Van Dam and Aleman, 2004; Kleinridders et al., 2014). Insulin not only regulates glucose and lipid metabolism in the brain, but also plays an important role in learning and memory (Zhao et al., 2004b). Insulin is actively transported across blood–brain-barrier (BBB) and it is also produced locally in the brain (Banks et al., 2012). Similar to insulin, IGF-1 is present also in rodent and human brain and can cross BBB (Duarte et al., 2012).

**Figure 1:**
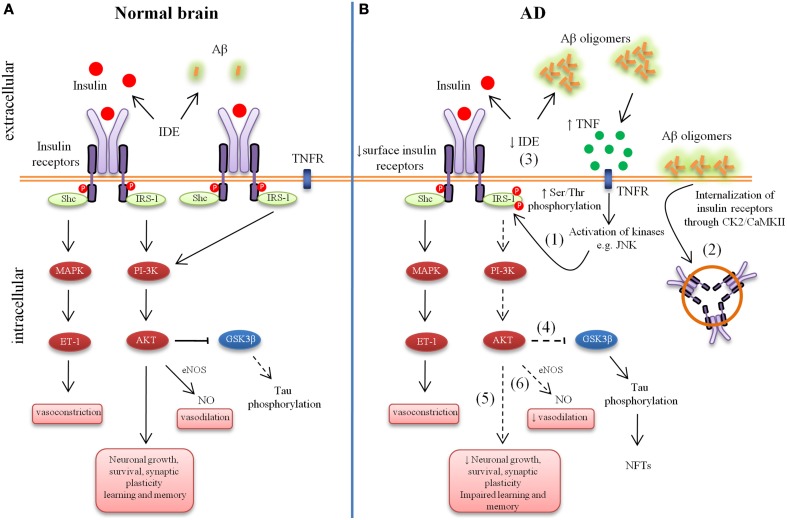
**Aberrant brain insulin signaling in Alzheimer's Disease (AD)**. Schematic outline of neuronal insulin signaling in the normal brain **(A)** and AD brain **(B)**. Under physiological conditions, insulin binding to its receptor triggers phosphorylation of insulin receptor substrate-1 (IRS-1). This results in phosphoinositide 3-kinase (PI3K) activation and downstream cellular responses that facilitate neuronal growth, neuronal survival, synaptic plasticity, learning and memory, etc. Activation of the IR can result in both vasodilatation and vasoconstriction and under physiological conditions there is a balance of both processes to regulate the immediate metabolic requirements of various tissues. In AD, accumulation of amyloid-β (Aβ) oligomers leads to increased tumor necrosis factor-alpha (TNF-α) levels and activation of stress kinases such as c-Jun N-terminal kinase (JNK) resulting in inhibitory serine phosphorylation of IRS-1 (1). Aβ oligomers cause removal of IRs from the cell surface mediated by Casein Kinase 2 (CK2) and Ca^2+/^Calmodulin-Dependent Kinase II (CaMKII) and redistribute them to cell bodies (2). Insulin resistance lowers the expression of Aβ-degrading insulin degrading enzyme (IDE) (3). Lowered IDE expression further decreases the availability of IDE for Aβ degradation. The reduction in brain insulin signaling increases GSK-3β activity (4), which increases abnormal tau phosphorylation. Deficient insulin signaling leads to impairment in nerve growth, synaptic plasticity, learning and memory, etc. (5). Aberrant phosphorylation of IRS causes an imbalance in homeostatic regulation of vascular function (6). This decreased production of NO may results in decreased cerebral blood flow and increased pro-inflammatory cytokines and reactive oxygen species production.

IR and IGF receptor type-1 (IGF-1R) are tetrameric glycoproteins that belong to the receptor tyrosine (Tyr) kinase superfamily, composed of two α and two β subunits (Schlessinger, 2000; Wada et al., 2005; Moloney et al., 2010; Duarte et al., 2012). Two different types of IR have been reported: neuron specific type (extensively expressed in neurons) and peripheral-like type (with lower density in glia cells) (Moreira et al., 2009). Due to structural and functional similarity, insulin and IGF-1 can activate both IR and IGF-1R, however they exhibit higher affinity to own receptors (<1 nM) (Conejo and Lorenzo, 2001). Once bound to their respective receptors, insulin or IGF-1 promotes autophosphorylation of tyrosin residue, triggering its intrinsic tyrosin activity and phosphorylating insulin receptor substrate (IRS) docking protein at tyrosine residue (Duarte et al., 2012). IRS-1 and IRS-2 are ubiquitously expressed and are the primary mediators of insulin-dependent mitogenesis and regulation of glucose metabolism in most cell types. IRS-1 was the first substrate identified and represents the prototype of the IRS family proteins, while IRS-2 was initially identified as an alternative substrate for the IR in animals with targeted disruption of the IRS-1 gene (Sesti et al., 2001). IRS is a critical switch in the insulin-signaling pathway, and also interacts with other receptor tyrosine kinases including IGF1/2, tropomyosin-related kinase receptor B (TrkB) and ErbB. The phosphorylation of IRS1 on tyrosine residues leads to the downstream activation of Akt, the mammalian target of rapamycin (mTOR) and glycogen synthase kinase 3 (GSK3), among other pathways; moreover, the phosphorylation of IRS1 on multiple serine (Ser) residues can inhibit IRS1 activity, leading to insulin resistance (Yarchoan et al., 2014). Several reports suggested that insulin signaling is altered in aging as well as in AD. Table 1 summarizes alteration of insulin signaling components in aging as well as in the AD. Figure 1 illustrates how aberrant insulin signaling is linked to AD pathology and how it forms a vicious cycle, which results in deterioration of learning and memory and neuronal loss. In the following section we will discuss the evidence of relation of insulin signaling deficiency with AD pathology and disease progression.

**Table 1:** **Altered insulin signaling in normal aging and Alzheimer's diseases**.

**Subjects/Tissue**	**Components of insulin signaling system**	**Observation**	**References**
**AGING**			
Rats/hippocampus	IGF-1 IGF-2	Unchanged [125I]IGF-I, [125I]IGF-II or [125I]insulin binding levels in normal aging process	Dore et al., 1997
Human patients/plasma	IGF-I	↓ plasma IGF-I levels with aging	Mustafa et al., 1999
Fisher 344 × brown Norway hybrid rats/plasma and CSF	IGF-1 IGF-1R IGFBP	↓ mRNA and protein levels in aged animals	Ashpole et al., 2015
Aged C57BL6 Mice/whole brain, serum and CSF	IGF-1	↓ IGF-1 levels in brain, CSF and serum in aged mice ↑ IGF-1R in aged mice hippocampus ↓ IGF-1R/Akt/GSK3 signaling pathway	Muller et al., 2012
**ALZHEIMER'S DISEASE**			
AD human patients/Hippocampus, cortex, and cerebellum	IGF-II Mannose -6-phosphate receptor	Unchanged	Kar et al., 2006
AD human patients/frontal, temporal, parietal, and occipital cortex	Insulin IR c-peptide levels	- Strong insulin immunoreactivity in pyramidal neurons compared to age-matched controls - Unchanged insulin and c-peptide levels - Increased IR density - No significant difference in IGF-1binding	Frolich et al., 1998
AD human patients/frontal cortex, hippocampus, hypothalamus	Insulin IGF-1 IGF-2 receptor mRNA	↓ IR and IGF-1R mRNA in hippocampus and hypothalamus ↓ insulin and IGF-2 mRNA in hippocampus and hypothalamus and IGF-1 in frontal cortex ↓ insulin, IGF-1, IR and IGF-1R-positive neurons in hippocampus ↓ IR and IGF-1R protein in hippocampus ↓ tyrosyl-phosphorylated IR, IGF-1R and IRS-1 in hippocampus, cortex and hypothalamus ↓ IRS-2 protein in hippocampus Impaired IRS-1 signaling in hippocampus and hypothalamus (↓p85-associated IRS-1) ↓ pAkt and pGSK-3b	Steen et al., 2005
AD human patients/Plasma, serum	IGF-1 IGFBPs	↑ total and unbound IGF-1 levels ↑ serum and CSF levels of IGF-1 and IGFBPs	Vardy et al., 2007; Salehi et al., 2008
AD human patients/CSF	Insulin	↓ insulin in mild AD patients ↓ insulin in MCI women - Positive correlation of insulin with Aβ_1−42_ levels and cognitive score	Gil-Bea et al., 2010
AD human patients/Temporal cortex	IGF-1R IGFBP-2	Unaltered IR protein levels however altered its distribution in AD neurons ↑ IGF-1R;↓IGFBP-2 ↑ IGF-1R surrounding and within plaques and in astrocytes ↓ IRS-1and IRS-2 protein levels ↑ phosphoIRS-1 levels near NFTs	Moloney et al., 2010
AD human brain tissue/ cynomolgus monkeys (i.c.v. injection of Aβ oligomer/AβPP-PS1 Tg mice	IRS-1	↑ IRS-1pSer636/639 levels in hippocampus ↑ IRS-1pSer636/639 levels were observed in neurons targeted by Aβ	Bomfim et al., 2012
AD human patients, triple transgenic mouse model, Cultured rat hippocampus neurons (Aβ oligomer insult)	IRS-1	↑ active JNK and IRS-1pSer616 levels Redistribution of IRS-1pSer616 expression from nucleus to cytosol in AD human patients and 3×Tg-AD ↑ IRS-1pSer616 colocalized with NFTs Aβ oligomer induced expression of IRS-1pSer616 in hippocampal neurons culture	Ma et al., 2009
AD human patients/cortex and hippocampus	Insulin IGF-1 IRS-1	- Unchanged basal levels of insulin and IGF-1 signaling molecules - Trend to increase IRS-1 levels in hippocampus - ↓ in responses to insulin signaling in the IR→IRS-1→PI3K signaling pathway and greater → in responses to IGF-1 in the IGF-1R→IRS-2→PI3K signaling pathway in AD - IRS-1 pS616 and IRS-1 pS636/639 correlated positively with Aβ plaques and negatively associated with memory	Talbot et al., 2012
AD human patients/frontal cortex	Insulin IGF-1 IRS-2	↓ insulin, IGF-1 and IGF-2 receptor mRNA and polypeptide mRNA in AD ↓ binding of ^125^I labled insulin, IGF-1 and IGF-2	Rivera et al., 2005
Hippocampal and cortical neuronal cultures S.D. Rats (soluble Aβ oligomer insult)	IR Tyr phosphorylation	Soluble Aβ oligomer inhibits IR activity (IR Tyr phosphorylation) ↑ Akt serine473 phosphorylation (Akt-pSer473)	Zhao et al., 2008
AD human patients/plasma	IGF-1	↓ plasma IGF-1 level in familial AD patients carrying the swedish AβPP 670/671 mutation	Mustafa et al., 1999
AD human patients/serum and CSF	IGF-1 IGF-2 IGFBP	↑ IGF-2 in both AD serum and CSF ↑ IGF-1 in AD serum ↑ IGFBPs in the CSF of the AD patients	Tham et al., 1993
AD human patients/CSF and plasma	Insulin	↓ CSF insulin and CSF-to-plasma insulin ratio ↑ plasma insulin	Craft et al., 1998
AD human patients/cortex	Insulin signaling	↓ total and phosphorylated components of insulin-PI3K-Akt signaling in AD	Liu et al., 2011
AD human patients/cortex and hippocampus	IRS-1	↑ IRS1-pS^616^, IRS1-pS^312^, Akt-pS^473^in AD Co-expression of IRS1-pS^616^ with pathological tau neurons	Yarchoan et al., 2014
AD human patients/plasma	Insulin	↑ plasma insulin after oral glucose tolerance test in AD patients ↑ CSF insulin levels	Fujisawa et al., 1991
Tg2576 mice of AD	IGF	↓ serum IGF levels	Carro et al., 2002

*IR, insulin receptor; IGF-1R, insulin growth factor receptor 1; IGF-2R, insulin growth factor receptor 2; IRS-1, insulin receptor substrate-1; IRS-1pSer636/639, IRS-1 phosphorylated at serine residues 636/639; JNK, c-Jun N-terminal kinase; PI3K, phosphatidyl inositol 3-kinase; CSF, cerebrospinal fluid*.

## Insulin signaling and AD pathology

Ample number of evidence suggested that alterations in brain insulin metabolism could be one pathological factor for neurodegenerative diseases including AD. In support of this hypothesis, AD patients have shown reduced brain insulin receptor sensitivity, hyperphosphorylation of insulin receptor and downstream second messenger such as insulin receptor substrate-1 (IRS-1) and attenuated insulin and insulin-like growth factor receptor expression (Watson and Craft, 2003; Rivera et al., 2005; Steen et al., 2005; Holscher and Li, 2010; Talbot et al., 2012; de La Monte, 2012c; Freiherr et al., 2013). Moreover, reduced CSF insulin levels have been observed in AD. However, few studies have demonstrated either normal or increased CSF insulin levels in AD patients (Freiherr et al., 2013). However, CNS has hyper-insulinemia in AD is still controversial.

### Neuritic (amyloid beta) plaques

The amyloid plaques found in the brains of patients with AD are mainly composed of Aβ, a peptide derived from a larger molecule that is known as the amyloid precursor protein (AβPP). AβPP is normally cleaved within its extracellular domain by a protease known as “α-secretase,” and the processing releases a large, soluble APP fragment (sAPPα) into the extracellular space (Querfurth and Laferla, 2010). Because cleavage occurs within the Aβ domain, α-secretase processing does not generate Aβ. Aggregation prone and damaging Aβ originate from proteolysis of the AβPP by the sequential enzymatic actions of beta-site amyloid precursor protein–cleaving enzyme 1 (BACE-1), a β-secretase, and γ-secretase (Querfurth and Laferla, 2010). Aβ peptides are natural products of metabolism consisting of 36–43 amino acids. Monomers of Aβ_40_ are much more prevalent than the aggregation-prone and damaging Aβ_42_ species. An imbalance between production and clearance, and aggregation of peptides, causes Aβ to accumulate, and this excess may be the initiating factor in AD (Querfurth and Laferla, 2010).

Several lines of evidence suggested a link between energy metabolism defects to functional alterations associated with pathogenesis of AD. Inhibition of energy metabolism can alter AβPP processing and induce amyloidogenic products (Gabuzda et al., 1994). The relationship between insulin and Aβ metabolism is receiving increasing attention. Recently, a direct link between the Aβ metabolism and insulin pathways has been described in neuronal cell lines (Solano et al., 2000; Gasparini et al., 2001). Insulin treatment elevated sAPPα secretion in a concentration- and tyrosin kinase-dependent manner in the neuronal cells and also reduced the Aβ accumulation in neuronal cells (Solano et al., 2000; Gasparini et al., 2001). It has been demonstrated that insulin and IGFs can protect neurons from Aβ-induced neurotoxicity in neuronal cultures (Mattson, 1997). Therefore, sAPPα derived from insulin-mediated metabolism of AβPP may function in modulation of neuronal excitability, synaptic plasticity, neurite outgrowth, synaptogenesis, and cell survival (Mattson, 1997). It has been recently shown that IGF-1 enhances clearance of brain Aβ by modulating transport and/or production Aβ carriers at the blood–brain interface in the choroid plexus (Ashpole et al., 2015). IGF-1 seems to increase Aβ clearance by enhancing transport of Aβ carrier proteins such as albumin and transthyretin into the brain (Carro et al., 2002). Under physiological conditions insulin degrading enzyme (IDE) is secreted at high levels from the microglial cells, and degrades Aβ extracellularly (Qiu et al., 1998). In addition to insulin degradation, IDE has been found to degrade Aβ in neuronal and microglial cell cultures, and to eliminate neurotoxic effects of Aβ (Qiu et al., 1998; Vekrellis et al., 2000; Sudoh et al., 2002). Insulin can modulate the extracellular degradation of Aβ through insulin degrading enzyme (IDE), which is involved in insulin catabolism (Kurochkin and Goto, 1994; Authier et al., 1996; Qiu et al., 1998; Farris et al., 2003). The expression of IDE is under control of insulin levels. Insulin via phosphatidylinositol-3-kinase (PI3K) pathway increases IDE protein levels (Zhao et al., 2004a). In line with this, IDE knockout mice exhibited elevated levels of cerebral Aβ (Farris et al., 2003). In contrary, overexpression of IDE in the AβPP double transgenic mice decreased their brain Aβ levels, and completely prevented Aβ plaque formation (Leissring et al., 2003). Further support for this link was provided by the findings that deficient insulin signaling (decreased PI3K subunit P85) was correlated with reduced IDE in AD brains and in Tg2576 Swedish AβPP transgenic mice (Zhao et al., 2004a). Aβ degrading activity of IDE was shown to be lower in AD brains compared to controls (Perez et al., 2000). On the other hand, Aβ oligomers cause rapid and significant disruption of signaling by brain cell IRs.

It has been demonstrated that, acute treatment with Aβ oligomer to hippocampus caused a rapid and substantial loss of neuronal surface IRs specifically on dendrites bound (Zhao et al., 2008; de Felice et al., 2009). Aβ oligomer-treated neurons showed elevated levels of IRs in their cell bodies, suggesting redistribution of IRs (Figure 1). The rapid redistribution of surface receptors rather than net receptor loss in short term Aβ oligomer treatment was further supported by Western blots, which showed no Aβ oligomer-induced changes in total IR levels (Zhao et al., 2008). This leads to decreased responsiveness to insulin, revealed by impaired insulin-induced receptor protein tyrosine kinase activity in cultured neurons exposed to oligomers (Zhao et al., 2008; de Felice et al., 2009). The Aβ oligomer-induced redistribution of IRs is consistent with the reports in which other synaptic proteins, such as NMDA receptors subunits and EphB2 receptor tyrosin kinase, also showed surface loss by soluble Aβ oligomers (Snyder et al., 2005; Lacor et al., 2007). Although Aβ oligomer-induced removal of NMDA receptors is mediated by α-7 nicotinic receptors, the Aβ oligomers-induced loss of dendritic IRs dose not follow similar mechanism (Snyder et al., 2005). Aβ oligomers are known to cause internalization of NMDA receptors through Casein Kinase 2 (CK2) and Ca2+/Calmodulin-Dependent Kinase II (CaMKII) (Chung et al., 2004). Therefore it was hypothesis that Aβ oligomer-induce internalization of NMDA receptors and IRs might share common mechanisms involving CK2 and CaMKII. Consistent with this hypothesis De Felice et al showed that Aβ oligomer caused major downregulation of membrane surface IRs via mechanism sensitive to CK2 and CaMKII (de Felice et al., 2009).

IRs play key roles in the important neurological processes including learning and memory and tau phosphorylation. Thus, Aβ oligomer-induced loss of membrane IRs might represent an important early mechanism underlying memory impairment and other pathological features of AD. Therefore, Aβ oligomers-induced loss of IR receptors can lead to learning and memory impairments and over tau phosphorylation. Further research in this field revealed that Aβ oligomers-induced neuronal oxidative stress. IR down-regulation and synaptic loss are markedly decreased by insulin treatment (de Felice et al., 2009). Neuroprotection by insulin requires IR tyrosin kinase activity, which suggested that insulin through IR might downregulate Aβ oligomer binding sites in the synapse instead of a simple competition between Aβ oligomers and insulin for a common binding site on the neuronal surface.

Inhibition of brain insulin signaling by streptozotocin, a compound known to induce diabetes, treatment to rat astrocytoma cells induced amyloidogenic protein expression as evidenced by the increase in AβPP, BACE-1, and Aβ_42_ expression (Rajasekar et al., 2014). Furthermore, it has been demonstrated that Aβ oligomer-induces elevation in proinflammatory tumor necrosis factor-alpha (TNF-α) levels and it triggers aberrant activation of c-Jun N-terminal kinase (JNK) in neurons, which ultimately leads to serine phosphorylation of IRS-1 (Bomfim et al., 2012). IRS-1 serine phosphorylation (IRS-1pSer) blocks the downstream insulin signaling, which triggers, in turn, peripheral insulin resistance (de Felice, 2013). Aberrant insulin signaling accelerates plaque production in the brain by enhancing the amyloidogenic processing of the AβPP (Wang et al., 2010; Son et al., 2012), and also increases Aβ aggregation through monosialotetrahexosylganglioside (GM1) clustering and membrane signaling (Yamamoto et al., 2012). Thus, Aβ oligomer-induced insulin resistance may create a vicious cycle in which oligomers upregulate their own production and aggregation by disrupting insulin physiological actions. Such a mechanism could account in part for Aβ oligomer build up in AD brains.

### Neurofibrillary tangles

Tau is a neuronal microtubule-associated protein found in axons. Tau plays an important role in assembly and stability of microtubules as well as in vesicle transport in neurons. Tau, in its hyperphosphorylated form, is the major component of paired helical filaments (PHFs), the building block of neurofibrillary lesions in AD brain. Hyperphosphorylated tau is insoluble, does not have affinity for microtubules, and self-associates into paired helical filament structure (Querfurth and Laferla, 2010). Abnormal hyperphosphorylation of tau prompts an accumulation of NFTs in axons of neurons, can impair normal axon transport, disrupt synaptic plasticity and finally induce cell loss (Querfurth and Laferla, 2010; Bedse et al., 2015). Evidence suggests that abnormal activation of kinases like GSK-3β as well as caspases may be responsible for hyperphosphorylation of tau (Churcher, 2006; Rohn, 2010). IR activation, through phosphorylated IRS proteins, results in activation of multiple signaling pathways including PI3K and extracellular signal-regulated kinase (ERK) that directly regulated various physiological processes (Figure 1) (Saltiel, 2001). Activation of PI3K→Akt cascade promotes neuronal growth and survival (Rodgers and Theibert, 2002). Akt inactivates GSK-3β, which inhibits tau phosphorylation (Zhao et al., 2009; Perluigi et al., 2014). There are number of evidence showing that insulin regulated tau phosphorylation and increased rate of NFT development (Hong and Lee, 1997; Lesort and Johnson, 2000; Schubert et al., 2003; Cheng et al., 2005; Freude et al., 2005). It has been shown that insulin and IGF-1 regulate tau phosphorylation through the inhibition of GSK-3β in cultural neurons. These effects of insulin and IGF-1 are mediated through the inhibition of GSK-3β via the PI3K-protein kinase B (PI3K-PKB) signaling pathway (Hong and Lee, 1997). Moreover, peripheral hyperinsulinemia promotes tau phosphorylation *in vivo* (Freude et al., 2005). When IGF-1 and IRS-2 gene were deleted, tau phosphorylation was dramatically increased in IGF-1 and IRS-2 knockout mice (Schubert et al., 2003; Cheng et al., 2005). IGF-1 genetic deletion specifically increased tau phosphorylation at two specific residues, Ser-293 and Ser-202, both GSK-3β targeted sites (Cheng et al., 2005). Inhibition of brain insulin signaling by intra cerebroventricular administration of streptozotocin also induces hyperphosphorylation of tau at multiple sites (Deng et al., 2009; Chen et al., 2013). This treatment dramatically increased total tau and hyperphosphorylated tau in the hippocampus of triple transgenic AD (3×Tg-AD) mice (Chen et al., 2013, 2014a). These results suggested that insulin and IGF-1 signaling normally prevents tau hyperphosphorylation in the brain. As mentioned earlier in the review, diabetes is characterized by insulin resistance, hyperinsulinemia and impaired insulin signaling. In type 2 diabetes increased GSK-3β activity might lead to an elevation of Aβ production (Phiel et al., 2003) and increased tau phosphorylation (Freude et al., 2005; Sims-Robinson et al., 2010). On the other hand, Aβ oligomer induced JNK activation leading to phosphorylation and degradation of the adaptor protein IRS-1 (Ma et al., 2009). IRS deficiency contributes to insulin resistance in diabetes. Significantly reduced IRS-1 and IRS-2 levels occur in AD brain, accompanied by elevated cytosolic phosphor-IRS1 (Ser 312 and 316) (Ma et al., 2009; Bomfim et al., 2012; Talbot et al., 2012; Yarchoan et al., 2014). Phosphorylation of IRS-1 (Ser 312 and 316) inhibits the regulation of insulin on GSK-3β activity, which leads to further increase in hyperphosphorylation of tau (Ma et al., 2009). The association of IRS1 abnormalities and tau was further supported by double immunofluorescence experiments demonstrating frequent co-expression of IRS1-pS616 with tau lesions in neurons and dystrophic neuritis (Yarchoan et al., 2014). The activity of GSK3 can be down regulated in response to insulin or IGF-1 through the activation of the PI3K pathway.

### Reduced cerebral blood flow

Neurons depend on blood vessels for their oxygen and nutrient supplies, and for the removal of carbon dioxide and other potentially toxic metabolites from the brain's interstitial fluid. Recent evidence suggests that vascular dysfunction leads to neuronal dysfunction and neurodegeneration, and that it might contribute to the development of proteinaceous brain and cerebrovascular “storage” disorders (Zlokovic, 2011). AD is characterized by a decreased regional cerebral blood flow that could result in decrease brain supply of oxygen, glucose, and nutrients. Insulin signaling regulates vasodilation and vasoconstriction (Aulston et al., 2013). IR activation mediates vasodilation through PI3K→Akt pathway. It stimulates endothelial nitric oxide synthase (eNOS) resulting in the production of nitric oxide (NO) and vascular relaxation (Figure 1A) (Bolotina et al., 1994; Kahn et al., 1997). IR activation can also mediate vasoconstriction. Activation of IR can also lead to phosphorylation of the Src homology containing (Shc) protein, which in turn binds the Growth factor receptor-bound protein 2 (Grb-2) resulting in the activation of Son of sevemless (Sos) protein. This complex then activates the Rat sarcoma (Ras) protein leading to phosphorylation of Rapidly accelerated fibrosarcoma (Raf) protein kinase that results in activation of Mitogen-activated protein kinase (MAPK). Activation of MAPK stimulates release of endothelin-1 (ET-1), a vasoconstrictor (Figure 1A) (Potenza et al., 2005, 2006; Formoso et al., 2006). By mediating vascular properties, insulin signaling plays an important role in glucose and oxygen availability to the brain (Aulston et al., 2013). In insulin resistant state, there is a specific impairment in the vasodilatory PI3K pathway, whereas the Ras/MAPK-dependent pathway is unaffected (Jiang et al., 1999; Cusi et al., 2000). This results in decreased NO production and increased production of ET-1 in humans leading to vasoconstriction (Figure 1B) (Piatti et al., 1996). NO further plays important role in protecting blood vessels from endogenous injury by preventing platelet and leukocyte interactions (Kubes et al., 1991; Sarkar et al., 1996). Decreased production of NO allows for increased expression of proinflammatory transcription of the nuclear factor-κB (NFκB), and production of chemokines and cytokines (Zeiher et al., 1995). The resultant decrease in nutrient availability to the brain due to decreased NO production results in an increase of oxidative stress and reactive oxygen species (ROS) production and an increased inflammatory response. Released pro-inflammatory cytokines and macrophage recruitment instigates the onset of atherosclerosis, ultimately leading to macrovascular complications (Aulston et al., 2013).

Moreover, insulin can improve dendritic spine density and rescue spine loss caused by apolipoprotein E 4 (APOE4). APOE4, a major genetic risk factor for AD, exerts neuropathological effects through multiple pathways, including impairment of dendritic spine structure and mitochondrial function. It has been shown that insulin sensitizers, such as rosiglitazone [peroxisome proliferator-activated receptor gamma (PPAR-γ)], significantly increased dendritic spine density and rescued detrimental effects of APOE4 on dendritic spine (Brodbeck et al., 2008). Thus, it is suggested that rosiglitazone might improve cognition in AD patients by increasing dendritic spine density. There is also evidence that insulin sensitizers modulate mitochondrial function (Wang et al., 2002). Fuenzalida and co-workers reported that rosiglitazone treatment in neuronal cells up-regulates Bcl-2 thereby stabilizing mitochondrial potential and protecting against apoptosis (Fuenzalida et al., 2007).

## Intranasal insulin delivery to the brain

Thus, impaired insulin sensitivity of IR and increased insulin resistance at brain may contribute to number of pathological processes that lead to acceleration of AD pathology. Therefore, restoring insulin to normal levels in the brain by insulin treatment, as showed in laboratory animals, may provide therapeutic benefits in AD subjects. Unlike, insulin cannot be administered orally, since it is degraded in the gastrointestinal track by various enzymes. Increasing brain insulin levels in AD patients by intravenous administration has been shown to acutely improve performance on hippocampus-dependent memory task (Craft et al., 1996). However, high systemic doses would be needed to achieve functionally effective insulin concentrations in the brain, causing strong peripheral side effects such as hypoglycemia and induction and/or exacerbation of peripheral insulin resistance (Benedict et al., 2011). Therefore oral and intravenous route of administration is not viable in clinical setting. In contrast, intranasal administration is a promising approach to selectively enhance brain insulin levels while avoiding adverse side effects (Born et al., 2002). This route offers a relatively high bioavailability, avoidance of the first-pass effect and invasive administration. Intranasal administration of insulin provides rapid delivery of insulin to the central nervous system via bulk flow via along olfactory and trigeminal perivascular channels, and slower delivery via olfactory bulb axonal transports (Thorne and Frey, 2001; Craft et al., 2012). Clinical studies have shown that intranasally administered insulin reaches to cerebrospinal fluid (CSF) within 30 min without substantial uptake into the bloodstream, bypassing the bloodstream (Born et al., 2002). Moreover, insulin exerts rapid changes on oscillatory electroencephalogram (EEG) parameters (Hallschmid et al., 2004). However, such comparable effects on EEG were not observed to those induced by intravenous bolus injection of insulin, suggesting that following intranasal administration, a significant amount of administered insulin dose reaches to the brain in a functional active state (Hallschmid et al., 2004). Recently, National Institute of Health (NIH) has selected intranasal insulin administration as one of the two therapeutic strategies receiving substantial funding as part of the National Alzheimer's Plan in the US (Wadman, 2012). This plan is a part of the initiative to find a therapeutic treatment to cure AD by 2025.

## Intranasal insulin improves memory in the healthy humans as well as in the AD patients

Enhancing central nervous insulin action has been shown to improve memory functions in animals as well as in humans (see Table 2) (Benedict et al., 2011). In a human study, the effects of 8 weeks of intranasal insulin (4 × 40 IU/d) and placebo were assessed on hippocampus-dependent declarative memory in 38 health subjects (Benedict et al., 2004). At the beginning and end of treatment, in total 30 words were orally presented to subjects. The subjects were asked to write down all words they still remembered after 3 min (immediate recall session) and one week later (delayed recall session) after presentation of words. The subjects treated with the insulin remembered significantly more words in delayed recall session, but not in immediate recall session, compared to placebo group. In this study the improving effect of subchronic intranasal insulin administration seemed to be specific for hippocampus dependent declarative memory, without any peripheral side effects. As far as the increased delayed recall authors pointed out that the improvement in the insulin-treated group occurred on a background of a generally decreasing performance. In addition, authors suggested that it might reflects an influence on encoding of the words at learning or a direct influence on retrieval, rather than an effect on the proper consolidation of memory. The same research group further studied effects of rapid-acting insulin analog insulin aspart, regular human insulin and placebo under similar experimental conditions (Benedict et al., 2007). Authors hypothesized that rapidly absorbed insulin aspart would show stronger effects on memory functions than regular human insulin due to the higher efficiency to reach brain. After 8 weeks of treatment subjects treated with both regular human insulin and insulin aspart remembered significantly more words than placebo group. Insulin aspart-treated subjects performed even better that those treated with regular human insulin (Benedict et al., 2007).

**Table 2:** **Intranasal insulin improves memory function**.

**Subjects**	**Intranasal insulin duration/dose**	**Main result**	**References**
Healthy humans	4 × 40 IU/day, for 8 weeks	Intranasal intake of insulin enhanced long-term declarative memory and positively affected mood in humans without causing systemic side effects like hypoglycaemia.	Benedict et al., 2004
Healthy humans	4 × 40 IU/day, for 8 weeks; insulin and rapid-acting insulin analog insulin aspart	Declarative memory was improved in insulin and insulin aspart groups compared to placebo group without altering glucose levels. Insulin aspart treated subjects performed even better that those of insulin treated group	Benedict et al., 2007
Healthy humans	Single dose of regular human insulin 160 IU	Hippocampus-dependent memory and working memory were improved in women where as men did not benefit from acute insulin treatment	Benedict et al., 2008; Krug et al., 2010
MCI and mild AD patients	20 or 40 IU of insulin acute treatment	Acute intranasal insulin administration improved verbal memory in AD and MCI subjects without the APOE- ε 4 allele	Reger et al., 2006
MCI and AD patients	10, 20, 40, or 60 IU for 5 days	10, 20, and 40 IU of insulin improved declarative memory only in APOE-ε 4 negative patients Memory facilitation generally peaked at the 20 IU dose ↑ Aβ42 levels for memory-impaired adults from saline to 10 IU regardless of APOE-ε 4 status Intranasal insulin did not affect peripheral glucose or insulin levels	Reger et al., 2008a
MCI and AD patients	20 IU BID intranasal insulin treatment for 21 days	Insulin-treated subjects retained more verbal information and improved attention and functional status Insulin treatment raised fasting plasma Aβ_40_/Aβ_42_ ratio	Reger et al., 2008b
MCI and mild to moderate AD patients	20 or 40 IU for 4 months	Treatment with 20 IU of insulin improved delayed memory Both dosages preserved caregiver-rated functional ability and general cognition Unchanged Aβ_42_ and tau levels after insulin treatment	Craft et al., 2012
AD patients with ApoE4 allels	Rapid acting insulin	Rapid acting insulin failed to have an acute impact on cognition in ApoE4 carriers with AD	Rosenbloom et al., 2014
MCI and mild AD patients	20 or 40 IU of insulin detemir for 21 days	High dose (40 IU) improved visuospatial and verbal working memory for all participant High dose improved memory for adults with MCI and AD who were APOE-ε 4 positive patients APOE-ε 4 carriers taking high dose also improved peripheral insulin resistance APOE-ε 4 negative patients taking high dose experiences increased peripheral insulin resistance	Claxton et al., 2015
S.D. rats—streptozotocin induced AD model	5 IU for 6 days	Insulin administration significantly reduced the Aβ levels without altering peripheral glucose levels	Subramanian and John, 2012
Streptozotocin induced-rat model of type 2 diabetes	2U insulin intranasally/4 weeks 6.7 U/kg (s.c.)/4 weeks	Decreased Akt activation and increased tau phosphorylation and GSK-3β activation was found in T2D rat brains. Intranasal insulin treatment normalized Akt and GSK-3β and reduced tau phosphorylation in diabetic rats s.c. insulin had minimal effect of tau phosphorylation and GSK-3β	Yang et al., 2013
3 × Tg-AD mice	1.75 U/7 days	Intranasal insulin administration restored insulin signaling, ↑ synaptic proteins, and ↓ Aβ40 level and microglia activation in the brains of 3 × Tg-AD mice. Glucose transporters and tau-phosphorylation is unchanged	Chen et al., 2014c
3 × Tg-AD mice	1.75 U/7 days Propofol 250 mg/kg (i.p.)	Insulin treatment attenuated propofol-induced hyperphosphorylation of tau, promoted brain insulin signaling. Resulted in down-regulation of several tau protein kinases, including cyclin-dependent protein kinase 5, calcium/calmodulin-dependent protein kinase II, and c-JunN-terminal kinase and up-regulation of protein phosphatase 2A	Chen et al., 2014b

In a study, gender differences regarding acute effects of central nervous insulin 160 IU were studied in 32 young healthy subjects (18 women and 14 men) in hippocampus-dependent two-dimensional-object location task and working memory task (digit span) (Benedict et al., 2008). Acute insulin treatment significantly improved hippocampus-dependent memory and working memory in women but not in men. The same group of researchers further showed that these effects were not restricted only to young women but also for postmenopausal women (Krug et al., 2010). Based on the encouraging effects of intranasal insulin that demonstrated improvement of memory without any side effects in healthy humans, few clinical trial have been carried out in patients with mild cognitive impairments (MCI) and AD patients. In a first study, 26 memory-impaired subjects (13 with early AD and 13 with mild MCI) and 35 normal controls received intranasal insulin (20 or 40 IU) or saline (placebo) treatments (Reger et al., 2006). Cognition was tested 15 min post-treatment, and blood was acquired at baseline and 45 min after treatment. Intranasal insulin treatment did not change plasma insulin or glucose levels. Insulin treatment facilitated recall on two measures of verbal memory in memory-impaired APOE negative adults. These effects were stronger for memory-impaired APOE negative subjects than for memory-impaired APOE positive subjects and normal adults. Unexpectedly, memory-impaired APOE positive subjects showed poorer recall following insulin administration on one test of memory. This study provided further evidence for APOE-related differences in insulin metabolism. Another study supported the same observation that intranasal administration improves cognition in APOE negative patients (Reger et al., 2008a). Recently, it has been demonstrated that high dose of insulin (40 IU) can improve visuospatial and verbal working memory in APOE positive MCI and AD patients (Claxton et al., 2015). High dose of insulin also improved peripheral insulin resistance in APOE positive patients. Conversely, high dose of insulin experienced increased peripheral insulin resistance in APOE negative patients (Claxton et al., 2015). These findings suggest that groups with different genetic risks for AD may show differential dose-response curves following intranasal insulin administration (Reger et al., 2008a; Rosenbloom et al., 2014). Further, it is reported that insulin treated subjects retained more verbal information, improved attention and raised fasting plasma Aβ_40_/Aβ_42_ ratio (Reger et al., 2008b). Another study supported the notion that the intranasal administration of insulin improves cognition in patients affected by mild cognitive impairment or AD (Craft et al., 2012). However, authors did not observe any changes in the Aβ_42_ and tau levels in the CSF after treatment.

## Conclusions

The advances in AD research in the last decade have revealed that AD is linked to insulin signaling deficiency in the brain. This hypothesis was further strengthen when intracerebroventricular administration of streptozotocin resulted in AD-like cognitive impairments, neurodegeneration and insulin resistance (Lester-Coll et al., 2006; de La Monte, 2014). Insulin/IGF signaling promotes the trafficking of AβPP-Aβ (Watson et al., 2003) and also enhances clearance of Aβ by modulating Aβ transporters and carriers at the BBB (Carro et al., 2002; Ashpole et al., 2015). Moreover, insulin/IGF negatively controls Aβ intracellular deposition, tau phosphorylation and degradation by IDE (Gasparini et al., 2001, 2002). Deregulation of insulin/IGF signaling increases Aβ deposition, tau phosphorylation, reactive species and decreases cerebral blood flow (Figure 1) (de La Monte, 2014). Accumulation of Aβ oligomers further worsen insulin deficiency by decreasing insulin's binding affinity to its receptors, reducing and desensitizing cell surface IRs and phosphorylating IRS-1 (Figure 1) (Lacor et al., 2007; Zhao et al., 2008; Ma et al., 2009; Fernandez and Torres-Aleman, 2012; Talbot et al., 2012). This review proposed intra nasal administration as potential therapeutics, which has been shown to improve cognition (Table 2) without any side effects in clinical trials.

### Conflict of interest statement

The authors declare that the research was conducted in the absence of any commercial or financial relationships that could be construed as a potential conflict of interest.
